# An American Text-Book of Physiology

**Published:** 1897-09

**Authors:** 


**An American Text-Book of Physiology.** Edited by WILLIAM H. HOWELL, M.D. Pp. 1052. Philadelphia: W. B. Saunders. London: The Rebman Publishing Company, Ltd. 1896.

Although the science of physiology has of late years grown so rapidly that a comprehensive text-book has become more and more difficult to produce, yet no work has been issued in England which is the outcome of the collaboration of several authors. It has been felt, probably, that in a subject the different branches of which are so closely connected, anything like diversity of style and treatment should be avoided. And it is no doubt better for many reasons that one mind should combine and reduce to order and significance the numberless physiological facts which have slowly accumulated. But it is hardly possible for a single individual to do this thoroughly. If the book is to be anything like a complete embodiment of modern knowledge, the author must either be content to write a somewhat condensed manual or bring out the work in successive volumes with the risk that vol. i. will be out of date by the time vol. iii. appears.

This American text-book is the joint work of ten teachers of physiology, all of them well known as professors in universities and medical schools. Purely anatomical and histological details are, for the most part, omitted, and attention is chiefly paid to the elementary facts of the science, and as a result we have a carefully compiled text-book of modern physiological knowledge. Here and there, as might be expected, special importance is given to some subject which an ordinary student or practitioner might deem unnecessary. This is the case, for instance, with parts of the very able chapter on the central nervous system. But we can hardly grumble at this when the matter given is so interesting.

The introduction, which is an essay on the meaning of physiology and an account of some of the vexed questions, such as "vitality," which are connected with it, is very well written; and we are glad to see that Professor Howell removes a stumbling block at the very outset. Protoplasm, he says, "must not be misunderstood to mean a substance of a definite chemical nature or of an invariable morphological structure; it is applied to any part of a cell which shows the properties of life, and is therefore only a convenient abbreviation for the phrase 'mass of living matter.'" In fact, throughout the entire volume great pains are taken to simplify matters as much as possible.

The chapter on Reproduction is up-to-date, and contains a good *résumé* of the modern views of heredity. But like most accounts in physiology books, it is hardly full enough to teach the uninitiated this complicated subject. This remark may also be applied to the chapters on the special senses; but the book had to be kept within certain limits, and it would be only too easy to make such a work of undue length.

The diagrams are numerous and valuable; and we would especially praise the excellent schemata and plans for elucidating the arrangement of the nervous system.

The style is generally good, and the editor is to be congratulated on the arrangement of the chapters and on the general organisation and appearance of the work.

